# Multi-Omics Reveals the Role of Osteopontin/Secreted Phosphoprotein 1 in Regulating Ovarian Aging

**DOI:** 10.3390/jpm14010078

**Published:** 2024-01-09

**Authors:** Li-Chuan Hsu, Chia-Jung Li, Li-Te Lin, Li-Fei Pan, Zhi-Hong Wen, Jim Jinn-Chyuan Sheu, Kuan-Hao Tsui

**Affiliations:** 1 Institute of Biomedical Sciences, National Sun Yat-sen University, Kaohsiung 804, Taiwan; taylor6174@gmail.com; 2 Department of Obstetrics and Gynaecology, Kaohsiung Veterans General Hospital, Kaohsiung 813, Taiwan; 3 Institute of Biopharmaceutical Sciences, National Sun Yat-sen University, Kaohsiung 804, Taiwan; 4 Department of Obstetrics and Gynaecology, National Yang-Ming University School of Medicine, Taipei 112, Taiwan; 5 Department of General Affair Office, Kaohsiung Veterans General Hospital, Kaohsiung 813, Taiwan; lfpan@vghks.gov.tw; 6 College of Finance and Banking, National Kaohsiung University of Science and Technology, Kaohsiung 824, Taiwan; 7 Department of Marine Biotechnology and Resources, National Sun Yat-sen University, Kaohsiung 804, Taiwan; wzh@mail.nsysu.edu.tw; 8 Department of Obstetrics and Gynecology, Taipei Veterans General Hospital, Taipei 112, Taiwan; 9 Department of Medicine, Tri-Service General Hospital, National Defense Medical Center, Taipei 114, Taiwan

**Keywords:** ovarian aging, bioinformatics, SPP1, spatial transcriptomics

## Abstract

Secreted phosphoprotein 1 (SPP1), also known as osteopontin (OPN), is located on chromosome 4q22.1. This multifunctional secreted acidic glycoprotein is expressed intracellularly and extracellularly in various tissues, where it interacts with regulatory proteins and pro-inflammatory immune chemokines, contributing to the pathogenesis of multiple diseases. Nevertheless, the intricate genetic connections between SPP1 and ovarian aging remain largely unexplored. This study aims to bridge this knowledge gap by delving into ovarian aging and its associations with SPP1 using multi-omics data analysis. Our findings indicate that SPP1 is a potential gene related to ovarian aging. To comprehend the role of SPP1, we conducted spatial transcriptomic analyses on young and aged female mouse ovaries, revealing a significant decline in SPP1 expression in the aging group compared to the young group. Similarly, a significantly low level of SPP1 was found in the 73-year-old sample. Additionally, in-depth single-cell RNA-sequencing analysis identified associations between SPP1 and ITGAV, ITGB1, CD44, MMP3, and FN1. Notably, co-expression analysis highlighted a strong correlation between SPP1 and ITGB1. In summary, this study pioneers the identification of SPP1 as a gene implicated in ovarian aging. Further research into the role of SPP1 has the potential to advance precision medicine and improve treatment strategies for ovarian aging-related conditions.

## 1. Introduction

The female reproductive system plays a crucial role in women’s health, with its significance extending across the lifespan. As women age, a natural decline in their reproductive capabilities occurs. This phenomenon persists even within assisted reproductive programs, where older women often encounter significantly lower pregnancy rates and implantation rates compared to their younger counterparts [1]. Delving into the causes and molecular mechanisms of ovarian aging becomes imperative for addressing age-related diseases and fostering women’s health and longevity. A pivotal aspect of female reproductive aging involves a gradual reduction in the quantity of human oocytes and a concurrent deterioration in oocyte quality. These factors contribute significantly to the overall decline observed in the reproductive capacity of women as they age. Understanding the intricate processes involved in ovarian aging is essential not only for reproductive health but also for developing strategies to enhance overall well-being and longevity in women.

Secreted phosphoprotein 1 (SPP1), commonly referred to as osteopontin, emerged initially as a pivotal sialic acid protein, intricately involved in bone physiology. Its role extended to facilitating the binding of osteoclasts to the calcified bone matrix [2]. Beyond its foundational role in bone health, SPP1 proves to be a versatile protein with far-reaching implications for various biological and pathological processes. These include active involvement in inflammation, immune responses, vascular remodeling, wound healing, and the intricate regulation of bone turnover [3]. Notably, SPP1’s association with cancer is profound, with its pivotal role in tumor invasion, metastasis, apoptosis, and angiogenesis documented extensively [4,5,6,7]. Shedding light on its significance in cardiovascular health, studies by Shen et al. reveal that suppressing SPP1 leads to improvements in cardiac function, prevention of cardiac remodeling, reduction in oxidative stress and inflammation, and inhibition of cardiomyocyte apoptosis [8]. Recent investigations have also underscored the importance of SPP1, a cytokine, in the retina and optic nerve head. It demonstrates its ability to protect retinal ganglion cells and preserve visual function in aging and glaucoma mouse models [9]. Furthermore, SPP1 has been implicated in the exacerbation of renal ischemia–reperfusion injury through the inhibition of the PI3K/AKT signaling pathway [10]. Despite the plethora of roles attributed to SPP1 across diverse physiological contexts, its impact on ovarian aging remains uncertain, adding an intriguing layer to its biological complexity. Consequently, the precise implications of SPP1 in the context of ovarian aging remain an enigma, prompting the initiation of this study to embark on a detailed exploration of SPP1’s role and influence within the intricate landscape of ovarian aging.

The primary objective of this study is to pinpoint potential biomarkers associated with ovarian aging, with a specific focus on SPP1. Employing a comprehensive approach that integrates single-cell RNA sequencing and spatial transcriptome analysis, we aim to thoroughly scrutinize the patterns of association among central genes across various data repositories. The outcomes of this investigation hold significant promise in two main domains. Firstly, it has the potential to unearth novel biomarkers that can enhance the diagnosis of ovarian aging, providing valuable insights for clinicians and researchers. Secondly, it can contribute to a deeper understanding of the crucial regulators of SPP1, potentially paving the way for more effective management and treatment strategies for this condition.

## 2. Materials and Methods

### 2.1. Analysis of Spatial Transcriptomics Data

Processing spatial transcriptomics data involves examining the patterns of gene expression and their spatial distribution in ovarian tissue sections derived from both young and elderly individuals. Our analysis relies on the spatial transcriptomic data extracted from the GSE188257 dataset, which was previously generated in a separate study. We conducted the data processing within the R programming environment, leveraging the Seurat package. Seurat is a versatile tool tailored for the analysis of single-cell and spatial RNA-seq data. To enhance our understanding of the data, we engaged in clustering annotation. This entailed the categorization of spatial cell clusters based on histological data from tissue sections stained with hematoxylin and eosin. This step enabled us to distinguish distinct cell types and regions within the ovarian tissue. To further refine the cluster annotation and pinpoint cell types with precision, we turned to CellChat for mapping. Cell markers, characterized by their specific expression in particular cell types, played a pivotal role. By examining the expression of these markers within clusters, we could more accurately assign cell types to each cluster. To gauge the prevalence and relative abundance of common cell types, we employed the Single-Sample Gene Set Enrichment Analysis (ssGSEA) algorithm. This algorithm assesses common cell types by considering the average expression of genes across different clusters. Clusters demonstrating high expression of multiple cellular markers are indicative of specific cell types. This multifaceted process allows us to derive comprehensive insights from the spatial transcriptomic data.

### 2.2. Analysis of Single-Cell RNA-Seq Data

The analysis of single-cell RNA-seq data was conducted in accordance with a methodology previously outlined [11], which is succinctly summarized below. This analysis encompassed the processing of the barcode matrix using Seurat. Low-quality cells were excluded, and the UMI count data were standardized. Highly variable genes were carefully selected, and batch effects were addressed through the implementation of the “RunHarmony” function. Cell clusters were identified using “FindClusters” and visualized via tSNE. Differential gene expression (DEG) was assessed, and subclustering was conducted following the same procedural framework. Moreover, we adhered to the established quality control and tSNE cluster generation methodologies within the GEO dataset GSE130664. To categorize cell types, well-known marker genes were employed, and correlations between cell clusters were probed through correlation analyses.

### 2.3. Analysis of SPP1 Expression in the Human Tissue Atlas

Delving into the expansive Human Protein Atlas (HPA) repository, the tissue atlas emerges as a pivotal segment, showcasing the representation of 17,288 proteins spanning over 44 diverse human tissues and organs. In the course of our investigation, we strategically harnessed the immense potential of HPA to curate and synthesize data concerning gene expression levels in healthy ovarian tissues. This dataset was meticulously sourced from 73 individuals, ensuring a representative spectrum across different age groups, specifically ranging from 20 to 49 years old. To fortify our analysis, we augmented this information with mRNA expression data sourced from three distinct whole-body datasets. These datasets encompass HPA-generated data, a collaborative effort with the Genome-based Tissue Expression (GTEx) consortium. The RNA-seq methodologies employed in this collaboration further enriched our understanding by providing comprehensive insights into gene expression patterns across various tissues and organs. This robust integration of data from multiple sources ensures a multifaceted exploration of gene expression in healthy ovarian tissues, contributing to a more nuanced and detailed examination of molecular signatures associated with ovarian aging.

### 2.4. Protein–Protein Interaction

STRING, a web-based database resource known as the Search Tool for the Retrieval of Interacting Genes, stands as a pivotal platform in the realm of molecular interactions. Offering a comprehensive blend of both experimental and predicted interaction information, it provides users with a wealth of data accompanied by confidence scores. In the intricate framework of STRING, proteins featured in an interaction network are visually represented as nodes, while the connections between any two proteins are elucidated as edges. These edges within the network encapsulate a diverse spectrum of interactions, encompassing both direct (physical) and indirect (functional) associations. The origins of these interactions span various repositories, including experimental sources and computational prediction techniques. Whether stemming from laboratory experiments or advanced computational algorithms, STRING aggregates a wealth of data to construct a detailed depiction of the interconnectedness of proteins. Notably, each edge in the network is annotated with a score, providing a quantitative measure that reflects the level of confidence in the interaction. This score serves as a valuable metric, indicating the likelihood of the interaction’s occurrence. Through this nuanced approach, STRING facilitates a comprehensive understanding of the molecular landscape, empowering researchers to navigate and interpret the complexities of protein interactions with a heightened level of confidence and precision.

### 2.5. Statistical Evaluation

The research outcomes were subjected to analysis through three separate experiments. The data are presented as mean ± standard error. Statistical significance was assessed using GraphPad Prism 8.0 in conjunction with Tukey’s post hoc test. A significance threshold of *p* < 0.05 was considered statistically significant.

## 3. Results

### 3.1. Spatial Transcriptomic Assessment of SPP1

**Figure 1:**
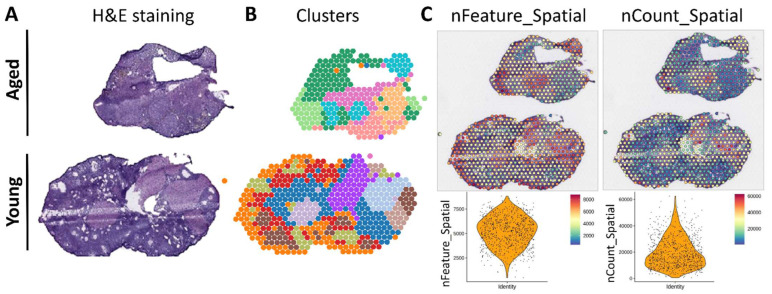
Spatial transcriptome examination of genetic alterations in aged and menstruating mouse ovaries. (**A**) Assessment of ovarian morphology through hematoxylin and eosin staining. (**B**) Superimposition of thirteen unsupervised spatial transcriptional histology clusters onto ovarian samples. (**C**) Evaluation of nFeature and nCount_spatial for quality and gene quantity.

In the initial phase of our research, we employed spatial transcriptomics—an advanced methodology that facilitates the precise mapping of transcriptomic patterns onto histological images of mouse ovaries stained with hematoxylin and eosin (H&E) (Figure 1A). This state-of-the-art technique enhances our comprehension of gene alterations in a two-dimensional space, offering a nuanced perspective on the spatial distribution and gene expression within the ovarian tissue at a single-cell resolution. To construct a thorough dataset, we utilized the 10× Visium platform. Subsequently, we employed unsupervised clustering and cell grouping via the Space Ranger software 2.0, revealing a total of 15 distinct clusters identified by their transcriptome characteristics (Figure 1B). To further explore cellular traits, we computed the number of genes per cell (nFeature), finding that a majority of cells exhibited a gene range of 5000–7000. Furthermore, the total Unique Molecular Identifier (UMI) count per cell exceeded 10,000 in most cells, providing a quantitative measure of gene expression intensity (Figure 1C). This in-depth analysis establishes the groundwork for a comprehensive understanding of the intricate molecular landscape within the ovarian tissue.

**Figure 2:**
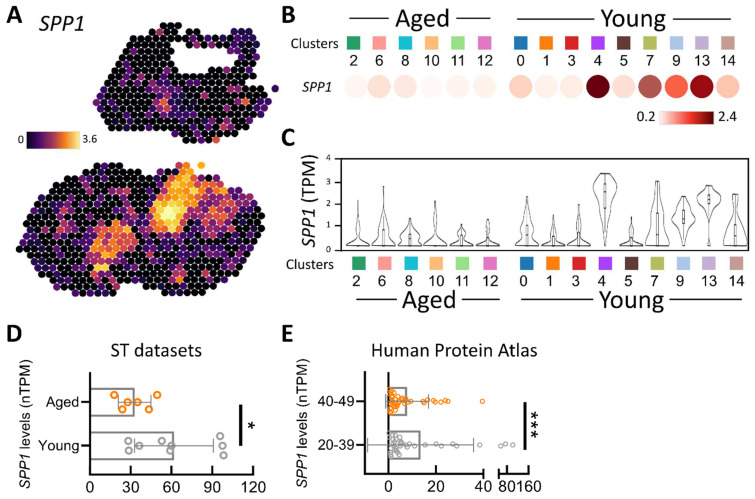
Spatial transcriptomic investigation of SPP1 expression and localization in mouse ovaries. (**A**) Visualization of the spatial distribution and genetic diversity of SPP1 in various tissue sections utilizing 10× Visium spatial gene representation analysis. Depiction of gene expression in dot plots and violin plots in (**B**,**C**), respectively, illustrating transcriptional activity across different clusters. (**D**) Evaluation of SPP1 gene expression in aged and young ovaries. (**E**) Human SPP1 expression in different age groups as displayed in the GTEx dataset. * *p* < 0.05, *** *p* < 0.001.

A comparative analysis of the two ovaries showed significantly higher SPP1 expression in the young group (Figure 2A). A more detailed examination of SPP1 levels within the 15 cell clusters was conducted, with dot plots vividly illustrating higher SPP1 levels in different cell clusters in the younger ovaries (Figure 2B). Figure 2C provides information on SPP1 transcripts per million and their distribution within each cell cluster. Upon quantifying these 15 cell clusters, SPP1 levels were significantly higher in young ovaries compared to old ovaries (Figure 2D). Additionally, we delved into human tissue databases to scrutinize SPP1 levels in the ovarian tissues of 73 women aged 20–49 years, revealing a notable difference between the 20–29 and 40–49 age groups. SPP1 levels in the 20–39 age group were notably higher than those in the 40–49 age group (Figure 2E). This comprehensive spatial transcriptome analysis provided insights into the spatial distribution of the SPP1 gene within ovarian tissue, particularly in the context of aging. It offers a detailed understanding of cellular composition and gene expression patterns in the ovarian microenvironment.

### 3.2. CellChat Reveals the Continuum of Cell Lineage SPP1-Related Signaling Events

**Figure 3:**
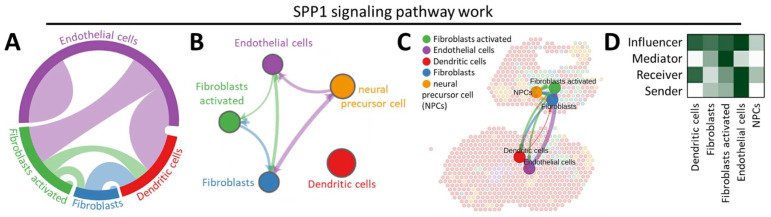
Interactions of the SPP1 signaling pathway with distinct ovarian cell types. (**A**) The circos plot illustrates the shared characteristics and correlations among various SPP1 cells within the ovary. (**B**) Visualization of cellular communication relationships at each level of the signaling pathway. (**C**) Cell–cell communication illustrates interactions between cells, with line thickness indicating the strength of these connections. (**D**) A heatmap displays the quantity of variations or the strength of interactions.

Employing CellChat, an in-depth analysis was conducted to unravel the intricacies of SPP1 signal interactions with diverse cell types. Our investigation initially focused on evaluating the quantity and intensity of these interactions within distinct cell subpopulations (Figure 3A) and subsequently comparing these interactions across the entire ovarian context (Figure 3B). The findings depicted in Figure 3B suggest a broad spectrum of variations in cell-to-cell communications within the comprehensive communication model. Following these assessments, cell populations within each signal transmission pathway were mapped into a weighted directed network. This approach facilitated the calculation of in-degree, out-degree, betweenness, and centrality, elucidating the roles of these cell populations—Receiver, Sender, Mediator, and Influencer—within the signal transmission pathway. Figure 3C illustrates our detailed analysis of intercellular communication specific to the SPP1 signaling pathway in the ovarian tissues of aging and young mice. Within the SPP1 signaling pathway, the principal signals of Sender, Receiver, and Influencer were primarily identified as endothelial cells, with fibroblast activation being the predominant signal (Figure 3D). This observation points towards the regulatory role of SPP1 signals in fibroblasts, endothelial cells, dendritic cells, and NPCs in the context of ovarian aging, highlighting the intricate network of cellular interactions orchestrated by SPP1.

### 3.3. Characterization of the SPP1 Gene in Ovarian Tissue by Single-Cell RNA Sequencing

**Figure 4:**
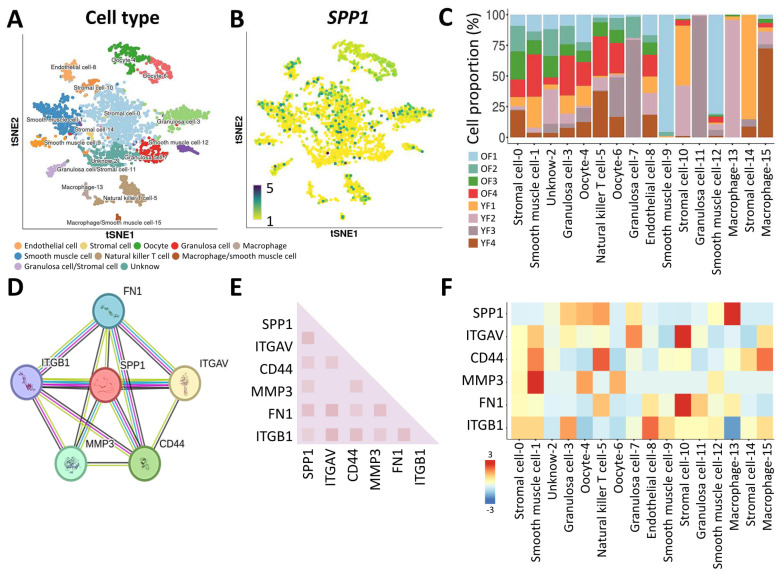
Evaluation of the expression of SPP1 in ovarian cells from individual primates. (**A**) tSNE plot depicting 15 distinct cell clusters. (**B**) Assessment of SPP1 expression levels and distribution across different cell types. (**C**) Quantification of cell counts and visualization of gene expression in diverse cell types. (**D**) Exploration of protein–protein interactions linked to SPP1. (**E**) STRING analysis investigating gene associations. (**F**) Heatmap analysis illustrates variations in SPP1-related hub genes within various cell populations.

Following this, our investigation shifted towards unraveling the transcriptomic landscape of the SPP1 gene concerning human ovarian aging, utilizing the Monkey Ovary Database (GSE130664). Rigorous cellular filtering was applied to this database, retaining high-quality transcriptomes from single cells (comprising 418 oocytes and 2183 somatic cells) collected from both young and old individuals (four each) for subsequent analysis. Our primary objective was to unveil the intricate cellular diversity within the ovarian microenvironment, as depicted in Figure 4A. Through meticulous elimination of batch-related variations and stringent quality control procedures, our analysis identified 16 significant cell populations, encompassing a total of 2601 cells, as illustrated in the tSNE plot. Our focus then shifted to the examination of the quantity and distribution of SPP1 transcripts across these 16 distinct cell populations, revealing a substantial presence of SPP1 in germ cells (Figure 4B). Initial profiling efforts were directed at characterizing different cell populations within ovarian tissue based on cell counts and RNA levels, as presented in Figure 4C. To gain a more profound insight into the functional relevance of SPP1, we delved into the protein–protein interaction network and conducted functional enrichment analysis using the publicly available STRING dataset. The outcomes uncovered associations between SPP1 and well-known genes such as ITGAV, CD44, MMP3, FN1, and ITGB1 (Figure 4D,E). Heatmaps were then employed to illustrate the expression levels of these genes in various cell clusters within ovarian cells, with particular emphasis on macrophages, where SPP1 exhibited heightened levels (Figure 4F). This comprehensive analysis provides a nuanced understanding of the role of SPP1 across diverse cell populations within the ovarian microenvironment.

**Figure 5:**
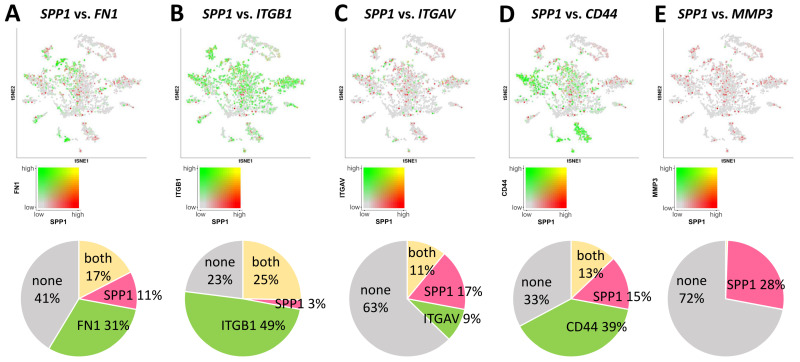
Evaluation of the connection between SPP1 and pivotal genes in various cell types. Investigation of the associations between SPP1 and different genes, such as FN1 (**A**), ITGB1 (**B**), ITGAV (**C**), CD44 (**D**), and MMP3 (**E**). SPP1 is indicated in red, while central genes are illustrated in green. The circular diagram below visually demonstrates the relative distribution of these two genes within this specific cell population.

Further delving into the intricate landscape of molecular interactions, our investigation aimed to unravel the nuanced associations between SPP1 and specific genes, embarking on a more thorough exploration. With meticulous scrutiny, we scrutinized the co-expression relationships between SPP1 (emphasized in red) and pivotal hub genes (highlighted in green) within the complex ovarian microenvironment. Notably, SPP1 exhibited co-expression with FN1 (31%), ITGB1 (49%), ITGAV (9%), CD44 (39%), and notably MMP3 (1%) in the same cellular milieu (Figure 5A–E). This substantial correlation, particularly the elucidation of the interaction between SPP1 and hub genes through the precision of single-cell RNA sequencing, brings to light potential regulatory connections. It hints at SPP1’s intricate involvement with genes closely linked to Integrin and the extracellular matrix. These insightful revelations bear significant importance, contributing to an enriched understanding of the molecular mechanisms that underlie ovarian aging and the associated physiological processes.

## 4. Discussion

Ovarian aging is a fundamental aspect of a woman’s reproductive journey, signaling a natural and inevitable progression [12]. This aging process is intimately tied to the gradual reduction in both the quantity and quality of oocytes, which are vital for fertility. One of the most noticeable consequences of ovarian aging is the diminishing reproductive capacity, leading to a decrease in fertility [1]. As women age, their chances of conception decrease, and the probability of experiencing difficulties in achieving pregnancy rises. This shift in fertility is often accompanied by a range of clinical symptoms, including endocrine imbalances and irregular menstrual cycles [1]. Understanding the dynamics of this aging process is essential for addressing age-related reproductive challenges and implementing timely interventions to support women through their fertility journey [13].

SPP1 has recently gained noteworthy prominence in cancer biology due to its direct association with tumor progression and its role in inducing immune cell infiltration [7,14,15,16]. In the realm of aging research, SPP1 has predominantly been explored within the context of neurological disorders. Prior studies have unveiled that SPP1 operates by promoting phagocytosis and the secretion of neurotrophic factors while concurrently inhibiting the production of neurotoxic and pro-inflammatory factors. SPP1 is also implicated in the upregulation of genes linked to oxidative phosphorylation, thereby enhancing mitochondrial respiration and fortifying the integrity of the mitochondrial microstructure. This leads to an elevation in intracellular ATP concentration through the upregulation of VDAC1 [9]. Furthermore, SPP1 has been associated with microglia activation and neuroinflammatory diseases. These genes and proteins have undergone extensive scrutiny in the cortical brain tissue of the elderly, particularly in the context of Alzheimer’s disease (AD) and related disorders [17]. The multifaceted role of SPP1 in the intricate landscape of cancer progression, neurobiology, and aging-related neurodegenerative conditions underscores its diverse and impactful contributions to physiological processes across different biological contexts.

Furthermore, studies in the field of reproductive medicine have revealed that the addition of dehydroepiandrosterone (DHEA) to decidualization hESF (human endometrial stromal fibroblasts) leads to increased expression of decidualization markers like IGFBP1 and PRL, as well as the endometrial permeability marker SPP1 [18]. This effect is most pronounced on day 8 of the decidualization process, coinciding with peak androgen concentrations [19]. Moreover, as we delve into the clinical implications of SPP1 in ovarian aging, it becomes evident that a comprehensive understanding of this molecular player could have transformative effects on fertility treatments and interventions. Considering the established role of SPP1 in promoting decidualization markers and endometrial permeability, its potential involvement in ovarian tissue remodeling and receptivity warrants exploration. This could pave the way for targeted therapies aimed at modulating SPP1 expression, potentially enhancing the reproductive environment in aging women.

There are currently no documented associations between SPP1 and ovarian aging. Nevertheless, previous studies have demonstrated that the absence or pharmacological inhibition of OPN, as well as bone marrow transplantation in OPN^-/-^ mice, can mitigate the ATM senescence-like phenotype, ultimately restoring healthy adipose tissue homeostasis in the context of aging [20]. The SPP1 gene, implicated in muscular dystrophy and bone loss, exhibits high expression in TREM2+ lipid-associated macrophages, suggesting its conserved role in age-related characteristics [21]. Similar findings in macrophages were observed in the analysis of intercellular communication data from single-cell RNA-sequencing data of skeletal muscle. This research reveals distinctive interactions between senescent fibroadipogenic progenitors and macrophages, involving mediators such as CCL2 and SPP1 [22]. SPP1 not only plays a crucial role in general age-related conditions but also engages with immune cells in cancer, influencing the immune microenvironment and impacting the progression of gastric cancer and pancreatic cancer [7,23]. These investigations underscore the pivotal role of SPP1 in immune-related aspects and offer fresh insights into prognostic markers and immunotherapeutic targets in aging and diverse diseases.

Our research team is devoted to unraveling the intricate molecular mechanisms that contribute to the aging of germ cells, driven by the overarching objective of pinpointing biomarkers for precise diagnostics and devising effective therapeutic strategies to enhance fertility in individuals navigating the challenges of ovarian aging [18,24,25,26,27,28]. In the course of this investigation, we embarked on a comprehensive analysis employing cutting-edge multi-omics approaches, incorporating state-of-the-art spatial transcriptomics and single-cell RNA-sequencing methodologies. Our primary focus centered on delving into the pivotal genes governing various germ cells within the context of ovarian aging, with a specific emphasis on understanding the role played by SPP1. Despite the valuable insights offered by our study, a myriad of pivotal questions remain unaddressed. It is imperative to illuminate the potential connections between SPP1 and the intricate cross-regulation of diverse germ cells within the ovarian microenvironment. Additionally, exploring the viability of harnessing SPP1 for the regulation of ovarian aging as a potential avenue for clinical therapeutic interventions remains an intriguing prospect for future research. It is noteworthy that the field of ovarian aging research is rapidly evolving, with its applications in the realm of reproductive health carrying immense practical significance for the management and treatment of associated conditions. We eagerly anticipate further exploration of these frontiers, foreseeing that it will not only expand our knowledge base but also make substantial contributions to the advancement of this critical field.

These findings not only shed light on the potential role of SPP1 in ovarian aging but also emphasize its broader significance in age-related conditions and its intricate involvement with immune cells in cancer. Understanding the role of SPP1 in immune-related aspects provides valuable insights into prognostic markers and potential immunotherapeutic targets in aging and various diseases. Further research in the clinical application of these findings could open new avenues for precision medicine and therapeutic interventions in the context of ovarian aging.

## 5. Conclusions

To summarize, our results emphasize the potential importance of SPP1 concerning the diagnosis and prognosis of ovarian aging. Utilizing comprehensive multi-omics analysis, we detected a significant reduction in SPP1 expression within aging ovaries. Furthermore, our investigation into the role of SPP1 and its interactions within the ovarian microenvironment suggests promising opportunities for interventions and precision medicine approaches in addressing concerns related to ovarian aging. Beyond its association with fertility and reproductive health, the intricate connections between SPP1, immune responses, and age-related diseases underscore its significance as a central player in the aging process. Further research and clinical applications of these insights hold the promise of not only enhancing our understanding of ovarian aging but also revolutionizing therapeutic approaches for women navigating the complexities of aging and reproductive health.

## Data Availability

The data presented in this study are publicly available in the GSE130664 and GSE188257.
